# Systematic review and meta-analysis of post-traumatic stress disorder as a risk factor for multiple autoimmune diseases

**DOI:** 10.3389/fpsyt.2025.1523994

**Published:** 2025-02-20

**Authors:** Kevin Mandagere, Savanna Stoy, Nathan Hammerle, Isain Zapata, Benjamin Brooks

**Affiliations:** ^1^ Rocky Vista University College of Osteopathic Medicine, Englewood, CO, United States; ^2^ Department of Biomedical Sciences, Rocky Vista University College of Osteopathic Medicine, Englewood, CO, United States; ^3^ Department of Biomedical Sciences, Rocky Vista University College of Osteopathic Medicine, Ivins, UT, United States

**Keywords:** PTSD, trauma, autoimmune disease, systemic lupus erythematosus (SLE), inflammatory bowel disease (IBD), rheumatoid arthritis (RA), multiple sclerosis (MS), psychoneuroimmunology

## Abstract

**Background:**

Post Traumatic Stress Disorder (PTSD) is a prevalent and debilitating psychiatric illness that has been linked to poor health outcomes and increased risk of developing chronic health conditions, including multiple autoimmune diseases such as Systemic Lupus Erythematosus (SLE), Inflammatory Bowel Disease (IBD), Rheumatoid Arthritis (RA), and Multiple Sclerosis (MS).

**Aim:**

This meta-analysis assesses the epidemiological research in this field and briefly explores the hypothesized neurobiological and immunological mechanisms that may underlie the association between PTSD and the development of Autoimmune Disease.

**Methods:**

PubMed, SCOPUS, and Cochrane Reviews databases were searched for all relevant articles in August 2023. Studies were systematically screened for relevance and inclusion criteria by two reviewers before quality assessment and data extraction were performed. Fixed and random-effect meta-analyses were performed to evaluate PTSD as a risk factor for the development of specific autoimmune diseases. Subgroup analyses examining the roles of biological sex and PTSD severity were also performed.

**Results:**

The initial search yielded 3010 articles where only eight prospective and retrospective cohort studies met criteria for inclusion in the meta-analysis. These eight studies were subdivided based on specific disease outcomes. Random effects model for risk of developing any autoimmune disease in persons with PTSD vs. control was 1.291 (95% CI = 1.179 to 1.412; P <0.001; n=1,984,310; 4 studies included). The strength of the association between PTSD and risk of developing specific autoimmune diseases varied by outcome condition from 1.142 (95% CI = 1.085 to 1.202, P <0.001) for risk of IBD to 1.302 (1.037 to 1.635, P = 0.023) for risk of MS. Random effects models showed statistically significant associations between PTSD and IBD, SLE, RA, MS, and Thyroiditis.

**Conclusion:**

These results suggest that the risk for developing autoimmune conditions, including SLE, MS, RA, and IBD, is significantly increased in the setting of PTSD. This association may have important implications on clinical practice and research into the pathophysiology of stress disorders.

## Introduction

Post-traumatic stress disorder (PTSD) is a psychiatric disorder recognized and diagnosed in the United States using the DSM-5 criteria for individuals aged six years and older (1). The diagnostic criteria are extensive and summarized as follows: direct exposure to actual or threatened death, serious injury, or sexual violence; presence of one or more intrusion symptoms associated with the traumatic event that begin after the traumatic event; persistent avoidance of stimuli associated with the traumatic event; negative alterations in cognition and mood following the traumatic event; marked alterations in arousal and reactivity following the traumatic event; symptoms must persist for longer than one month; symptoms must cause clinically significant distress and impairment in functioning; and the disturbance cannot be related to the psychological effects of a substance or other medical condition (1). PTSD is common in the United States, with a lifetime prevalence of PTSD among men of 5% and among women 10.4% (2).

PTSD causes significant morbidity, with war-related PTSD alone accounting for millions of disability-adjusted life years (DALYs) globally (3). PTSD has also been associated with social consequences such as disruptions of intimate relationships and decreased educational and professional outcomes, as well as a high burden of medical and psychiatric comorbidities, including cardiovascular disease, substance use disorder, and somatic symptom disorders (4, 5). A growing body of evidence from large, robust cohort studies has also implicated PTSD as a risk factor for the development of autoimmune conditions ranging from Systemic Lupus Erythematosus to Inflammatory Bowel Disease (IBD) (6–9). Several studies have also observed that PTSD severity correlates with a proportionally increased risk of developing autoimmune disease (6–8). While the overall association between PTSD and the development of autoimmune diseases as a whole appears relatively consistent across cohort studies, the strength of association and significance of the association of PTSD with specific autoimmune conditions, particularly Rheumatoid Arthritis (RA), IBD, and uncommon conditions such as Polymyositis, has been inconsistent (6, 7). Further analysis is needed to evaluate these inconsistencies and examine the potential for bias and confounding, such as population genetics, rates of smoking, and substance use (which are common comorbidities with PTSD) among different study populations (e.g., US Veterans vs. Swedish female civilians).

Recent studies have also begun to characterize the biological underpinnings of traumatic stress’ effects on the immune system. Traumatic stressors produce remarkable changes in both the innate and adaptive immune systems, as well as the neuroendocrine and autonomic nervous systems that regulate them (10–12). These include increased levels of proinflammatory cytokines, including CRP, IL-6, and TNF-α, and upregulation of the pro-inflammatory NF-κB transcription pathway. These effects are reinforced by reductions in anti-inflammatory cytokines such as IL-10 and dysregulation of the hypothalamic-pituitary-adrenal (HPA) axis, a key regulator of inflammation. Glucocorticoid levels and glucocorticoid receptor sensitivity are abnormally low in individuals with PTSD, disrupting an essential anti-inflammatory mechanism. Furthermore, hyper-sympathetic states lead to increased levels of catecholamines like norepinephrine, which directly affect monocytes and effector T-cells, promoting the production of pro-inflammatory cytokines and the upregulation of proinflammatory transcription factors, including NF-κB (12).

Research has also suggested that pro-inflammatory states may themselves be risk factors for the development of PTSD (12). These findings suggest a complex interrelationship between stress, immunity, and neurobiology. There is a growing body of evidence suggesting that pro-inflammatory states can lead to increased blood-brain barrier permeability and alterations in neurotransmitter levels, including glutamate, serotonin, and dopamine. Pro-inflammatory states may also modulate the function of brain regions implicated in PTSD, such as the insula, amygdala, and hippocampus (12). Of note, there is also recent, preliminary clinical evidence suggesting that glucocorticoid therapy in the acute phase following exposure to traumatic stress may reduce the development and severity of PTSD (13). Taken together, these findings suggest that the immune system and neurobiology are intimately connected and can be profoundly disrupted by traumatic stress (12).

This review aims to clarify the association between PTSD and a spectrum of autoimmune diseases, assess possible sources of bias and confounding, discuss future directions for epidemiological research in this field, and integrate pathophysiology research from the rapidly expanding field of psychoneuroimmunology.

## Methods

### Protocol

This systematic review and meta-analysis was conducted following PRISMA guidelines. The initial literature search was conducted using DistillerSR (DistillerSR Inc., Ottawa, Canada) literature review and analysis software.

### Inclusion/Exclusion criteria

For inclusion, studies must be: 1) Prospective or retrospective cohort analysis 2) Participants of studies must be >18 years of age 3) Examine diagnosed PTSD as an exposure and at least one diagnosed Autoimmune Disease (including but not limited to SLE, IBD, RA, MS, Type I Diabetes, or Thyroiditis) as an outcome. Exposure and outcome must be physician diagnosed or use a validated self-report tool; non-validated self-report data on exposure or outcome was cause for exclusion. 4) Provide quantitative data on exposures and outcomes in tables or **Supplementary Material** 5) Papers must be published in English 6) Papers must be of high or moderate quality, assessed via the CASP checklist. Papers found to be of low quality or with high risks of bias using the CASP checklist were excluded.

### Search databases

PubMed, SCOPUS, and Cochrane Reviews databases were searched for all relevant articles in August 2023. The scope of this search included all articles published before the search date. The results of these searches, including abstracts, were imported into DistillerSR.

### Search strategy/quality appraisal

Search terms included: Stress Disorder AND Autoimmune Disease; (PTSD OR Post Traumatic Stress Disorder) AND Autoimmune Disease; (PTSD AND IBD) OR (PTSD AND SLE) OR (PTSD AND Lupus) OR (PTSD AND RA) OR (PTSD AND UC) OR (PTSD AND Chrons) OR (PTSD AND MS) OR (PTSD AND Multiple Sclerosis) OR (PTSD AND Type I Diabetes) OR (PTSD AND Thyroiditis).

A total of 3010 articles from the search terms included below underwent initial screening in DistillerSR software. Two researchers independently screened each title and abstract to decide their initial relevance for the study. Then, the two individual researchers completed together a secondary and tertiary screening step using full texts to determine inclusion or exclusion based on designated criteria. Any disagreements on inclusion were resolved by discussion and consensus of all reviewers, including a third reviewer, independent screening records were not compiled. After three screening levels, two researchers categorized all articles meeting inclusion criteria based on study outcomes (i.e., autoimmune diseases). Studies were jointly appraised and rated for quality using the CASP Cohort Studies Checklist to assess validity, results, and potential impact of results. Disagreements were settled via consensus discussion.

### Data extraction

Three researchers, working independently, manually extracted data from each included study. Two separate reviewers working together then confirmed the accuracy of all extracted data prior to data analysis. The data extracted was related to the outcome of focus in this paper, the subsequent development of autoimmune disease following PTSD. Additional variables considered for data analysis were biological sex and severity of PTSD based on reported symptom burden.

### Statistical and bias analysis

Data extracted from each eligible study was evaluated through a meta-analysis using the Generic Inverse Variance method. This method was selected based on the ratios data available across studies. Eligible studies did not all evaluate the same variables therefore the studies included in each meta-analysis varied across models. Random effects models were used evenly in our interpretations for all meta-analysis presented even when heterogeneity (I^2^) was not significant in some instances. Fixed effect estimates are available in the **Supplementary Files**. Publication bias was assessed using both Egger’s and Begg’s test. Funnel plots for each analysis were developed in each assessment and are included in the **Supplementary Files**. All meta-analysis were performed in MedCalc v.22.023 64-bit (MedCalc Software Ltd., Ostend, Belgium). Significant differences are reported in all instances at a confidence level of 95%.

## Results

**Figure 1:**
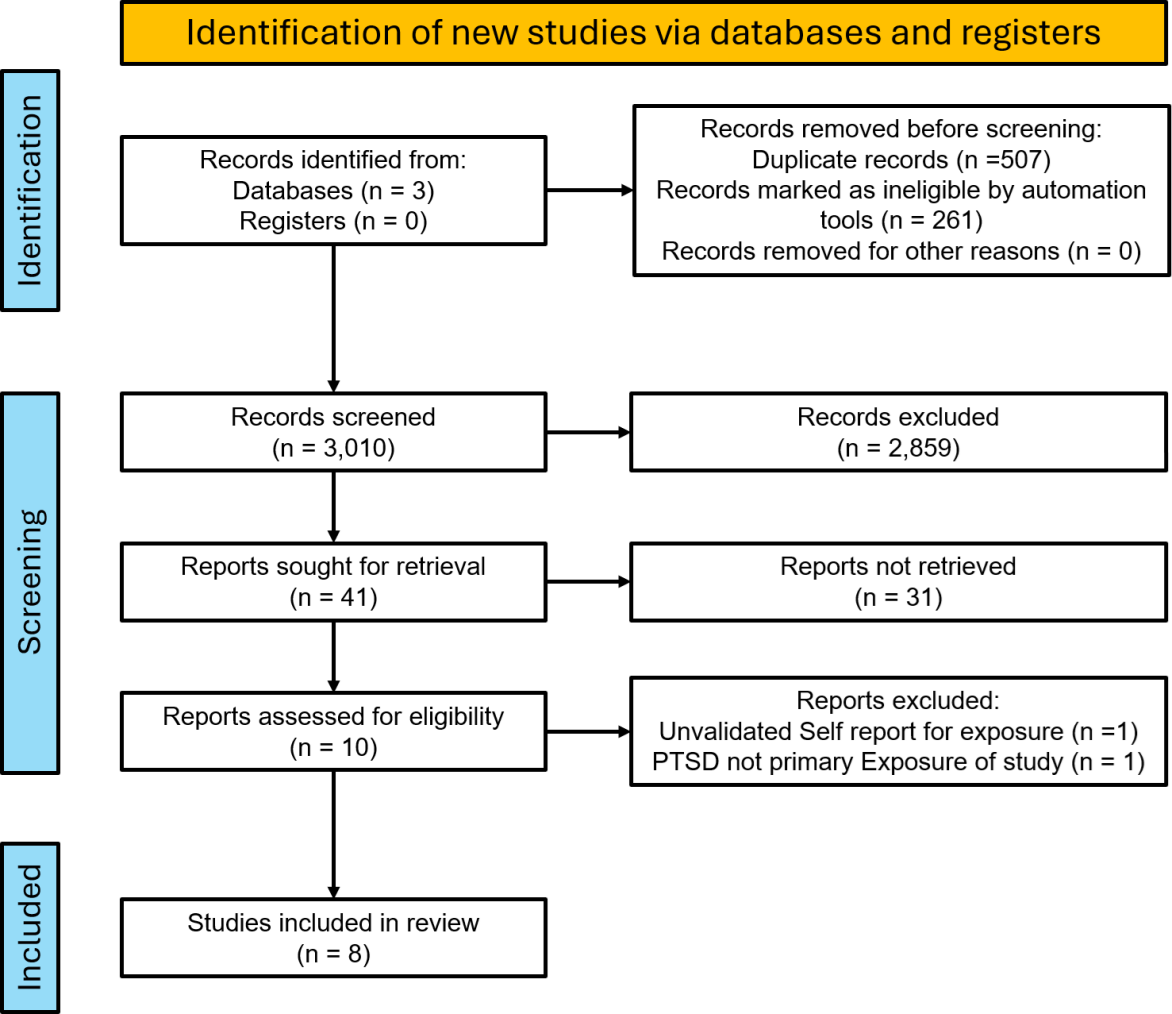
PRISMA flow diagram of the selection process for studies of PTSD and risk of autoimmune disease.

The initial search yielded 3010 articles after the removal of duplicates for initial screening. Title screening for relevance led to the exclusion of 2859 articles. A total of 41 studies were then abstract screened, leading to the exclusion of 31 based on unvalidated self-report metrics for diagnosis of AD, study design other than prospective or retrospective cohort, or focus on non-AD primary outcomes. 2 studies were later excluded on full-text review, 1 for reliance of unvalidated self-report of exposure and outcome and the other for study design and PTSD as a secondary exposure (**Figure 1**).

Ultimately, 8 cohort studies that met all inclusion criteria were identified for inclusion and classified as high or moderate quality using the CASP checklist (**Table 1**). These studies assessed heterogeneous populations, including United States Veterans, United States Nurses, and large cohorts of Swedish and Taiwanese citizens. Study sizes ranged from 45,992 to 1,171,104 and assessed a wide array of autoimmune disease outcomes. Criteria for PTSD exposure fell into two categories: Bookwalter, Jung, Lee, and Roberts used screening tools based on DSM-IV criteria, while O’Donovan, Song, Dai, and Hsu used ICD-9 codes. ICD-9 Codes represented the application of DSM-IV or V criteria by clinicians. Based on the dates of publication for these studies, DSM-IV was likely the predominant guideline utilized in their datasets.

**Table 1:** Overview of studies meeting inclusion criteria.

Author, year	Title	Study Population	Design	Sample Size	Persons at risk with condition	Persons at risk without condition	Primary Exposure	Outcomes	Stratification by Severity
O'Donovan, 2015 (8)	Elevated Risk for Autoimmune Disorders in Iraq and Afghanistan Veterans with Posttraumatic Stress Disorder	Iraq and Afghanistan veterans <55 y/o who were enrolled in Department of Veterans Affairs health system between October 7, 2001, and March 31, 2011.	Retrospective cohort	666,269	203,766	462,503	PTSD (n=203,766) and non-PTSD psychiatric disorders	Any Autoimmune Disorder, Thyroiditis, IBD, RA, MS, SLE	No
Lee, 2016 (16)	Posttraumatic Stress Disorder and Risk for Incident Rheumatoid Arthritis	Subset of female nurses from Nurses' Health Study II	Prospective Cohort	54,224	9,762	39,931	PTSD (n=9,762), Stratified by severity	RA	Yes
Roberts, 2017 (9)	Association of Trauma and Posttraumatic Stress Disorder With Incident Systemic Lupus Erythematosus in a Longitudinal Cohort of Women	Subset of female nurses from Nurses' Health Study II	Prospective Cohort	54,763	15,778	34,464	PTSD (n=15,778), Stratified by severity	SLE	Yes
Song, 2018 (6)	Association of Stress-Related Disorders With Subsequent Autoimmune Disease	Swedish National Inpatient Register (1964-present) and the Swedish National Outpatient Register (2001-present).	Retrospective cohort	1,171,104	106,464	1,064,640	PTSD (n=106,464), analysis used accumulated person-years at risk (n=911,730)	41 autoimmune diseases, Any Autoimmune Disease	Yes
Jung 2019 (21)	Posttraumatic stress disorder and incidence of thyroid dysfunction in women	Subset of female nurses from Nurses' Health Study II	Prospective Cohort	45,992	8,639	37,353	PTSD (n=8,639), Stratified by severity	Thyroiditis (Hypothyroidism and Graves' Hyperthyroidism)	Yes
Bookwalter 2020 (17)	Posttraumatic stress disorder and risk of selected autoimmune diseases among US military personnel	Millennium Cohort Study Active-Duty US Military)	Prospective Cohort	120,572	9,875	110,697	PTSD (n=9,875)	RA, SLE, MS, IBD, Any autoimmune disease	No
Dai, 2021 (15)	Posttraumatic Stress Disorder and the Associated Risk of Autoimmune Skin Diseases	National Health Insurance Research Database in Taiwan	Retrospective Cohort	49,005	9,801	39,204	PTSD (n=9,801)	Psoriasis, Lichen Planus, Alopecia Areata, Autoimmune Bullous diseases, and vitiligo	Yes
Hsu, 2023 (7)	Risk of autoimmune diseases after post-traumatic stress disorder: a nationwide cohort study	National Health Insurance Research Database in Taiwan	Retrospective Cohort	26,365	5,273	21,092	PTSD (n=5,273)	Any autoimmune disease, thyroiditis, SLE, RA, Sjogren's syndrome, IBD, Dermatomyositis and polymyositis	No

**Figure 2:**
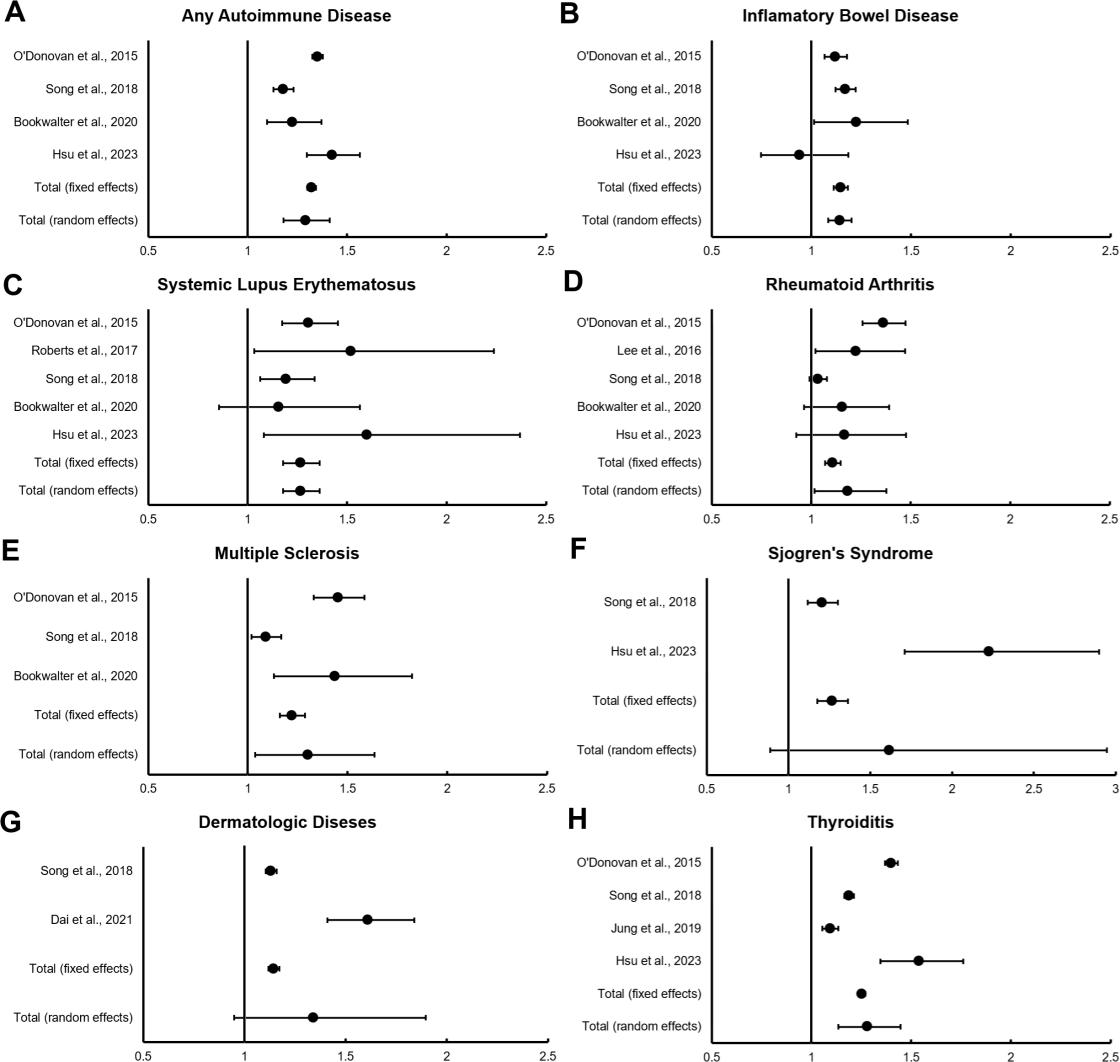
**(A–H)** Forest plots for meta-analysis of PTSD and risk of various autoimmune diseases.

Individual studies reported results as either Hazard Ratios or Risk Ratios. For our analysis, risk ratio estimates were generated for each study using the Generic Inverse Variance meta-analysis method. All studies reported strong, statistically significant associations between PTSD and multiple incident autoimmune diseases (**Supplementary Table S1**). A random effects model of the risk of developing any autoimmune disease was 1.291 (95% CI = 1.179 to 1.412; P <0.001) (**Figure 2A**). The strength of the association between PTSD and specific autoimmune diseases varied by outcome condition (**Figures 2B–H**). On the lower end, 1.142 (95% CI = 1.085 to 1.202, P <0.001) for a random effects model of PTSD and risk of IBD to 1.302 (1.037 to 1.635, P = 0.023) for PTSD and risk of MS on the highest end. Random effects models showed statistically significant associations between PTSD and IBD, SLE, RA, MS, and Thyroiditis.

**Figure 3:**
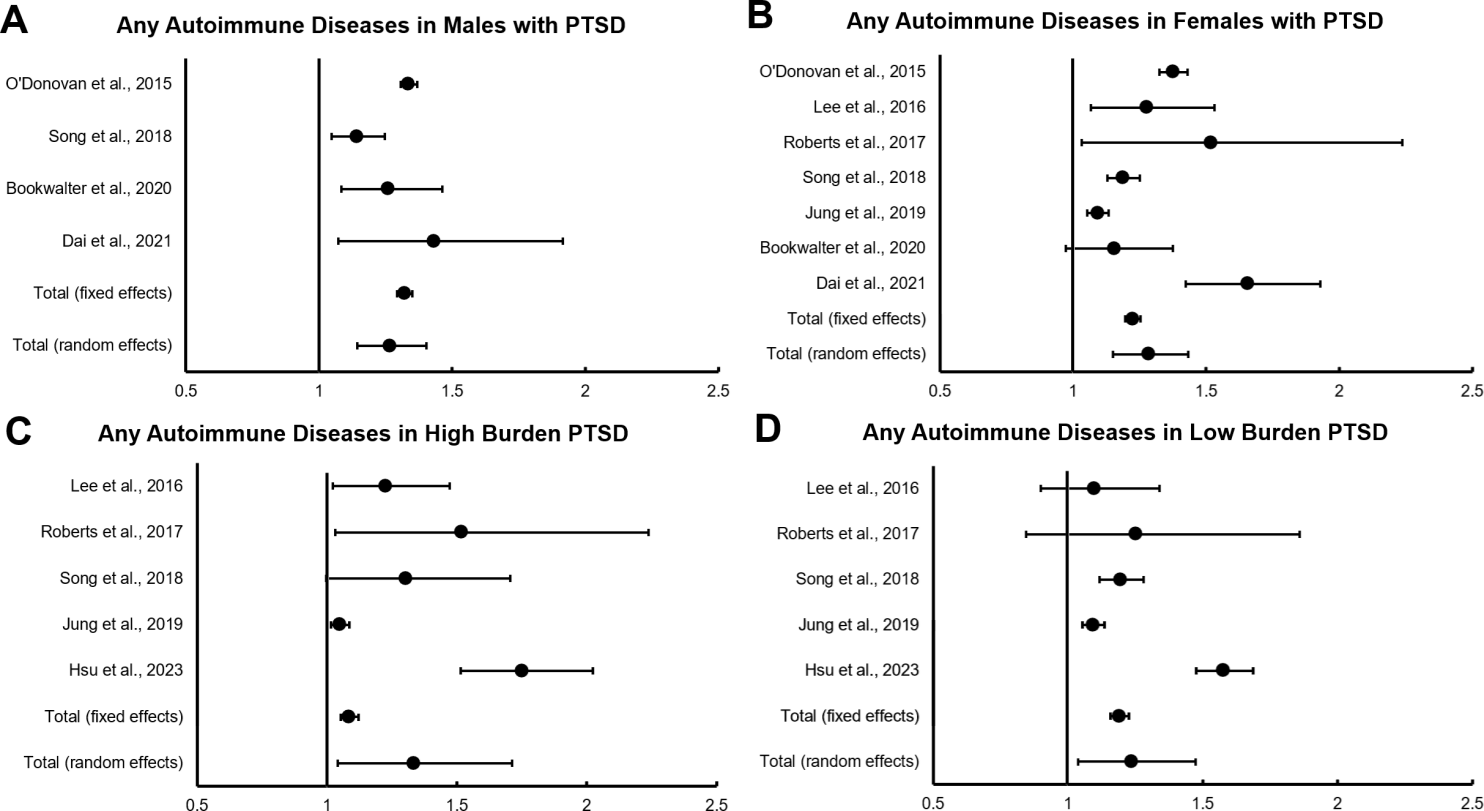
**(A–D)** Forest plots for subgroup analyses of PTSD and risk of autoimmune diseases.

Subgroup analyses (**Figures 3A–D**) examining the risk of developing autoimmune disease in male vs. female patients, men and women showed similar levels of increased risk: RR of 1.266 (95%CI = 1.143 to 1.403 P < 0.001) and RR of 1.285 (95%CI = 1.151 to 1.434 P < 0.001) respectively. For subgroup analyses of PTSD symptom severity, high symptom burden was associated with an RR of 1.335 (95% CI = 1.041 to 1.712; P = 0.023) while low symptom burden was associated with an RR of 1.236 (95% CI = 1.037 to 1.473; P = 0.018).

Results for heterogeneity analyses were statistically significant for several of the analyses and are available in the **Supplementary Files**. Only random effect models were used in our main discussion to maintain assumption consistency across models, fixed effect estimates are available in the **Supplementary Files**. Of note, our calculated estimates of association were often lower than those originally reported because our estimates were based on estimates rather than counts. However, our overall results and results by the individual outcomes were still significant and always in accordance with the findings in the original studies.

## Discussion

### Interpretation of results

This analysis adds to the growing body of evidence that PTSD is associated with the risk of developing a wide range of autoimmune diseases. Strengths of this analysis include its inclusion of diverse, mainly high-quality, heterogeneous cohort studies with large sample sizes. Most of these studies drew from datasets with robust demographic and medical information and, in aggregate, examined the effects of important potential confounding factors. These include smoking status, comorbid psychiatric illness, and family history. To our knowledge, this is the first meta-analysis to explore this association.

The risks of developing specific autoimmune diseases appear unequally distributed, with illnesses such as SLE and MS more often associated with PTSD compared to IBD and RA. It is unclear if this reflects differences in the pathogenesis of these illnesses or if these findings are attributable to population-level or demographic factors. These findings are consistent with the results of individual studies in our sample, including O’Donovan, Song, and Hsu (6–8). Results for an association between PTSD and the development of Sjogren’s and Dermatologic illnesses did not meet statistical significance in the random effects model, although both had significant associations in the fixed effect model. This may reflect the small number (n=2) of studies examining these conditions.

Subgroup analysis on risks of developing an autoimmune disease among male and female PTSD sufferers aligned with results of individual studies, which found that increased risk among males and females was proportional, although female PTSD sufferers are at overall greater risk due to the underlying association between female sex and autoimmunity (8, 14). Interestingly, results from the high and low symptom burden subgroups both showed significant increases in relative risk, but the difference between the two groups was relatively modest and not statistically significant. This may reflect the varied methodologies used by individual studies to categorize PTSD severity and our analysis’ limitation of only “high” and “low” symptom groups, as opposed to more granular sub-categorizations performed in some of the component studies. For example, Hsu and Song used measures of psychiatric care or resource utilization such as hospitalizations versus outpatient treatment only, while others, such as Lee, Roberts, and Jung, used symptom-based questionnaires to quantify severity (6, 7, 9, 15, 20).

### Limitations of evidence and review process

There are several important limitations in the evidence base. First, several studies (O’Donovan, Dai, and Hsu) were unable to control for the potentially confounding effects of tobacco and alcohol use in their analyses (7, 8, 15). This may explain why these studies frequently found stronger associations than other studies like Song and Bookwalter (**Supplementary Table S1** **–** **Supplementary Materials**). It is, however, worth noting that among the majority of studies that were able to control for smoking, it was found to have a limited impact on the observed association (16, 17). There was also an inconsistent adjustment for study participants’ race - where present, this was limited to white vs. nonwhite. This is despite differential rates of exposure to both trauma and autoimmune disease among specific racial groups, including African Americans (18, 19).

Second, studies were largely unable to assess or control for the impact of childhood trauma, which has itself been associated with a wide range of negative health and psychosocial outcomes (10, 20). Study populations were also entirely adults (>18 years of age), with PTSD diagnoses received in adulthood. Stratification by trauma type and exposure to previous trauma was primarily done in military cohort studies, which assessed combat exposure and military sexual trauma (8, 17). Third, multiple studies raised concerns regarding accurately assessing the temporality of the association given the long diagnostic lag time present in many autoimmune diseases. Several studies ran reverse-causality analyses examining if autoimmune diagnosis can increase risk of subsequently developing PTSD, but did not find evidence of this association (6, 8, 18, 21). Fourth, increased autoimmune disease detection among PTSD patients may be an artifact of their increased healthcare utilization (including for non-autoimmune disease medical illnesses) and screening. This was difficult to control for or assess across these cohorts, and only one study directly adjusted for health-care utilization by controlling for the number of primary care visits per year (17). Song et al. also attempted to correct for this possibility of surveillance bias by excluding the first year of follow-up in all of their analyses. Fifth, studies using the Nurses’ Health Study II relied on a validated-self report of physician diagnosed autoimmune disease and may therefore be at greater risk of biases such as differential response bias or recall bias than the investigations that used documented physician-diagnosed outcomes.

The reduced number of studies available for each synergistic analysis are a limitation of our study. For example, only 2 studies examined polymyositis or dermatologic conditions, limiting their statistical power. Analysis was also limited by the data reported in the included studies, complicating statistical analysis. Few studies reported the overall number of cases for each outcome, and some studies reported adjusted relative risk, while the majority reported hazard ratios. Relative to individual studies’ reported hazard ratios and relative risk, this appears to have shifted our results toward the null hypothesis. Furthermore, studies used a variety of methods to quantify PTSD severity, ranging from symptom rating scales to inpatient hospitalizations. For the purposes of our subgroup analyses focused on PTSD severity, we were forced to simplify these metrics into high vs. low symptom burdens. This may explain the lack of a dose-dependent effect in our analysis that was observed in several of the studies such as Jung and Lee, which used symptom severity scores rather than indirect metrics like hospitalizations (16, 21).

### Implications

Although the findings were statistically significant, effect sizes were modest, with the relative risk of developing any autoimmune disease at 1.291 (95% CI 1.179 to 1.412) for persons with PTSD. While this is a notably increased relative risk, the absolute risk of developing an autoimmune disease because of PTSD is likely relatively modest. Song et al. provides an estimate of 9.1 and 6.0 cases per 1000 person-years for PTSD and unexposed, respectively. This suggests that the yield for empirical screening for autoimmune diseases based solely on PTSD diagnosis is likely low. However, when examined within the overall context of the literature, which has also associated PTSD with both cardiovascular and metabolic disease, it is clear that special consideration to the medical needs of PTSD sufferers is imperative (5, 22, 23). Clinicians should therefore be aware of this association and be vigilant for signs that PTSD patients may be developing or suffering from autoimmune illnesses to reduce diagnostic delay. Counseling patients on the association between a PTSD diagnosis and physical illnesses may also be validating for patients and of additional psychological benefit.

It has been proposed that negative health outcomes in PTSD patients are due to changes in lifestyle (increased substance use, sleep disruptions, poor diet, etc.). Only a minority of studies were able to assess the role of these factors in the development of autoimmune disease. Bookwalter et al. attempted to address this question by examining the effects of smoking, “body size,” and alcohol use and found these covariates only minimally affected risk estimates for the development of autoimmunity (17). No studies have been able to directly examine the role of sleep, dietary, and social disruptions caused by PTSD in the development of autoimmune disease.

Further research into the association between traumatic stress and autoimmunity may benefit both our understanding of the pathogenesis of these illnesses and shed light on prevention and effective management. While Song et al. reported that “persistent use of SSRIs throughout the first year after diagnosis was associated with attenuated risk of autoimmune disease,” this result has not, to our knowledge, been further explored or replicated. Furthermore, research should address the social covariates that could contribute to the development of autoimmune disease, as it may help elucidate the underlying etiology of the association between traumatic stress and autoimmune disease development.

Future research topics of interest include the possible role of glucocorticoid therapy in the prevention and management of PTSD or the effects of trauma-focused therapies such as EMDR on perceived symptom severity in patients with PTSD and comorbid autoimmune illness.

### Conclusion

The risk for developing autoimmune conditions, including SLE, MS, RA, and IBD, is significantly increased in the setting of diagnosed PTSD. Other autoimmune conditions, including Sjogren’s and dermatologic illnesses, require further evidence to support an increased risk in the setting of diagnosed PTSD. Increased risk appears to be comparable in men and women, although women overall have higher rates of both PTSD and autoimmune disease. Although more severe PTSD symptoms did result in increased risk of developing an autoimmune disease, our analysis did not detect a clear dose-response relationship. This analysis adds to a growing body of evidence for PTSD’s role as a major risk factor for chronic medical conditions and has significant implications for our understanding of traumatic stress’ effects upon the immune system.

## Data Availability

The original contributions presented in the study are included in the article/**Supplementary Material**. Further inquiries can be directed to the corresponding author.
